# Tolerance to combined drought and heat stress in edamame is associated with enhanced antioxidative responses and cell wall modifications

**DOI:** 10.1111/ppl.70187

**Published:** 2025-03-27

**Authors:** Jeremiah M. Hlahla, Mpho S. Mafa, Rouxléne van der Merwe, Makoena J. Moloi

**Affiliations:** ^1^ Department of Plant Sciences‐Botany Division University of the Free State Bloemfontein South Africa; ^2^ Carbohydrates and Enzymology Laboratory (CHEM‐LAB), Department of Plant Sciences‐Botany Division University of the Free State Bloemfontein South Africa; ^3^ Department of Plant Sciences‐Plant Breeding Division University of the Free State Bloemfontein South Africa

## Abstract

Drought and heat stress often co‐occur in nature, and their combined effects are a major driver of crop losses, causing more severe damage to plant metabolism than when they occur individually. This study investigates the responses of three edamame cultivars (AGS429, UVE14, and UVE17) to combined drought and heat (DH) stress, with emphasis on the reactive oxygen species (ROS), antioxidative mechanisms and cell wall modifications. Malondialdehyde (MDA), electrolyte leakage (EL), and hydrogen peroxide (H_2_O_2_) were used to measure oxidative stress and membrane damage. The non‐enzymatic (ascorbic acid, AsA) and enzymatic (superoxide dismutase, ascorbate peroxidase (APX), guaiacol peroxidase, and glutathione reductase) antioxidant responses were determined spectrophotometrically. Cell wall biomass composition (cellulose, hemicellulose, lignin, and phenols) was determined using Fourier transform Infrared Spectroscopy and spectrophotometry. Ascorbate peroxidase activity and AsA content in DH‐stressed AGS429 at flowering strongly correlated to reduced lipid peroxidation (r^2^ = −0.97 and − 0.98). Cultivar UVE14 accumulated high AsA under DH stress at both growth stages, which, in turn, was positively associated with total phenolic content (r^2^ = 0.97), APX activity, and holocellulose, suggesting enhanced ROS‐dependent oxidative polymerisation. On the contrary, poor ROS quenching in UVE17 led to MDA accumulation (*p* ≤ 0.05), leading to high EL and poor cellulose synthesis at pod‐filling (r^2^ = −0.88). Therefore, at the physio‐biochemical level, AGS429 and UVE14 showed DH stress tolerance through enhanced antioxidative responses and cell wall modifications, while UVE17 was susceptible. Identifying the key biochemical traits linked to DH stress tolerance in edamame offers novel insights for breeding more resilient edamame cultivars.

## 1 INTRODUCTION

The impacts of climate change, exacerbated by anthropogenic activities, lead to elevated average temperatures and changes in rainfall patterns, all of which have adverse effects on agriculture (Idris et al., 2022). Edamame (*Glycine max* L. Merr.) is a nutrient‐rich legume, i.e., it has a high protein content, vitamins and minerals, making it an important crop for addressing malnutrition in developing countries. Although edamame originates from East and Southeast Asia (Nair et al., 2023), its global cultivation is increasing due to nutritional and economic benefits. For instance, Tanzania leads edamame's production in Africa with 71 varieties, followed by South Africa with 42 varieties (Djanta et al., 2020). However, the production of this crop is limited under changing climatic conditions such as heat and drought (Nair et al., 2023). Li & Zhang (2022) showed that the optimal soil temperature range for edamame seedling emergence is between 25 and 32°C. In addition, Van der Merwe et al. (2018) reported a significant reduction in edamame yield under drought stress. Furthermore, Chen et al. (2021) showed that although drought stress generally reduced pod dry weight, some cultivars had better resistance than others.

Future predictions suggest that global edamame production may decline by 25% in 2080 due to heat, causing excessive drought stress (Das et al., 2017). Cohen et al. (2021) found that combined drought and heat stress greatly reduce crop yields by lowering the harvest index, shortening the crop's growth period, and changing the number, size, and makeup of seeds. These effects happen because drought and heat stress disrupt the normal metabolic balance in plants, leading to reduced plant development and yield (Das et al., 2017). Separately, drought or heat stress in plants affects the process of photosynthesis, nutrient metabolism, absorption, translocation of ions, cellular respiration, carbohydrate metabolism, plasma membrane and cytoskeletal structural damage (Nair et al., 2023; Sharma et al., 2023). Most studies focus on drought and heat stress separately, however, considerable crop losses in the field could result from the combined stress (Balfagón et al., 2018). Moreover, the combined drought and heat (DH) stress could be more damaging, resulting in high lipid fluidity, reduced cell turgor pressure, poor cell development and early tissue senescence (Sharma et al., 2023). Individually, drought or heat stress, or their combined effect, exacerbate the production of reactive oxygen species (ROS), including singlet oxygen (^1^O_2_), hydrogen peroxide (H_2_O_2_), superoxide radicals (O_2_^−^) and hydroxyl radical (**·**OH) (Sekmen et al., 2014; Zhou et al., 2019).

A high buildup of ROS leads to oxidative stress in plant cells. This damages the cells by triggering the breakdown of membrane lipids, photosynthesis pigments, DNA, and proteins, ultimately hindering plant growth and development (Bhaduri & Fulekar, 2012; Rajput et al., 2021).

The extent of this damage can be assessed by measuring malondialdehyde (MDA), a by‐product of unsaturated phospholipid peroxidation, which serves as an indicator of membrane damage under environmental stress (Sekmen et al., 2014; Zhou et al., 2019). For example, an increase in H_2_O_2_ caused MDA accumulation due to membrane damage in two tomato cultivars under DH stress (Zhou et al., 2019). In addition, other studies reported a significant correlation between H_2_O_2_ accumulation and lipid peroxidation in cotton under DH stress (Sekmen et al., 2014).

To cope with the effects of oxidative stress induced by accumulation of ROS under drought, heat or DH stress, plants use enzymatic and non‐enzymatic ROS scavenging mechanisms. The enzymatic ROS scavengers include but are not limited to superoxide dismutase (SOD, EC 1.15.1.1), guaiacol peroxidase (GPOX, EC 1.11. 1.7), glutathione reductase (GR, EC 1.8.1.7) and ascorbate peroxidase (APX, EC 1.11.1.11); while the non‐enzymatic antioxidants include ascorbic acid (AsA) and phenols (Gill & Tuteja, 2010; Moloi & van der Merwe, 2021; Zainy et al., 2023). The O_2_^−^ radical is produced in the chloroplasts, peroxisomes and mitochondria and is disproportionated by SOD to produce H_2_O_2_, which through the action of catalase or peroxidase, is neutralised to form water (Indo et al., 2015; Del Río & López‐Huertas, 2016; Doğru, 2021). DH stress tolerance in citrus involves the regulation of ROS detoxifying enzymes, i.e., SOD, APX, catalase (CAT), and GR (Zandalinas et al., 2017). Li et al. (2021) observed an increase in antioxidant enzyme activity under drought or heat stress, but the combined drought and heat stress led to a decreased antioxidant enzyme activity, which was accompanied by high ROS and malondialdehyde (MDA) content in yellow horn (*Xanthoceras sorbifolium*). Zandalinas et al. (2017) argued that the accumulation of ascorbic acid (AsA) under DH stress in citrus was due to adverse environmental stress. In addition, AsA rapidly reacts with ^1^O_2_, H_2_O_2_, O_2_^−^ and ozone (O_3_), reducing their potential to cause oxidative damage in plant cells (Akram et al., 2017).

Alongside this defence mechanism, the structure of the cell wall is equally crucial for enhancing plant resistance to abiotic stress (Le Gall et al., 2015). Plants under abiotic stress, such as drought, adjust their cell walls to reduce shoot growth while maintaining root growth, utilizing ROS and peroxidases (Tenhaken, 2015). Plant cell walls are composed of structural polysaccharides, with microcrystalline and amorphous cellulose (β‐1,4‐glycosidic linkages), as well as hemicellulose, composed of xylans, mixed β‐glucans‐linkage, xyloglucans and mannans (Schädel et al., 2010; Mafa et al., 2020). Lignin forms cross‐links with hemicellulose through the ester bonds, which re‐enforces the cell walls and gives it a characteristic of stiffness (Le Gall et al., 2015; Mafa et al., 2020). The stiffness and rigidity of the cell wall are vital for plant response to environmental stress, including DH stress (Le Gall et al., 2015). A decrease in cellulose with an increase in callose due to DH stress was observed in cotton (Hu et al., 2022), while an increase in glucuronic acid was observed under DH stress in date palm (*Phoenix dactylifera*) (Safronov et al., 2017). The findings of these studies confirm that combined DH stress modifies the cell wall (Safronov et al., 2017; Hu et al., 2022). In addition, previous findings showed a reduction in the hemicellulose contents of the susceptible edamame cultivars (Hlahla et al., 2022), supporting the claim that cell wall modification assists plants in adjusting to environmental changes.

While the ability of plants to scavenge ROS and accumulate cell wall sugars has been recognized as a tolerance mechanism against various environmental stresses (Bachiri et al., 2020; Sekmen et al., 2014), little is known about how these processes function in edamame under combined drought and heat (DH) stress. To address this gap, this study investigated the effects of DH stress on ROS accumulation, enzymatic and non‐enzymatic antioxidative responses, and cell wall modifications in three edamame cultivars. The findings of this study provide valuable insights into the biochemical traits that contribute to edamame's tolerance, offering a foundation for pre‐breeding strategies aimed at improving resilience to DH stress.

## 2 MATERIALS AND METHODS

### 2.1 Plant material and growth conditions

The seeds of three edamame cultivars (AGS429, UVE14, and UVE17) were germinated in moist filter paper‐lined petri dishes at 20°C. The cultivars were selected based on their responses to drought stress. For example, AGS429 is drought tolerant with low yield reduction under drought stress; UVE14 is drought tolerant but is not a high‐yielding cultivar; and UVE17 is a high‐yielding cultivar that is susceptible to drought stress. After radicle emergence, the seedlings were transferred to the seedling tray (one seedling per cell) containing hygromix seedling mix (Hygrotech). The seedlings (one per pot) were transplanted in potting bags at the unifoliate leaf stage (vegetative one, V1 stage). The potting bags contained dried red sandy‐loamy soil (10 kg) watered to 100% soil water holding capacity (WHC; 11.6 kg). The plants were grown in the glasshouse (29°6′30.8” S; 26°11′19.4″ E) of the University of the Free State at 28°C (day) and 18°C (night). At the second trifoliate leaf stage (V3), plants were subjected to growth under a combination of drought (30% WHC) and high‐temperature stress (35/27°C day/night) (DH stress) throughout the experiment. Negative control plants were continuously watered to 100% WHC at 25/18°C (day/night). Positive control plants were subjected to either drought stress (30% WHC) or high temperature stress (35/27°C, day/night). The 35°C temperature and 30% WHC were chosen as suitable to cause combined drought and heat (DH) stress in edamame because the optimal temperature for photosynthesis ranges between 25–30°C, an increase of 32.8–35.6°C led to a huge yield loss in edamame (Onat et al., 2017) and severe drought stress (0–40%, WHC) caused a significant reduction in the rate of photosynthesis in edamame (Laxa et al., 2019; Ribas‐Carbo et al., 2005). The experimental design was a split‐split‐plot randomized complete block design (RCBD) with temperature as the main plot, the water treatment as the subplot, and the cultivar as the sub‐subplot. Three replications (with three pots/replication) per treatment were used. Leaf samples were collected on young, fully expanded trifoliate leaves at flowering and pod‐filling stages. Sampling was performed between 10:00 AM and noon when the sun was at its peak because the rate of photosynthesis was high between 8:00 AM and noon in Rose (*Rosa hybrida*) plants (Ibrahim et al., 2017). Young, fully expanded leaves were collected and crushed in liquid nitrogen and stored at −26°C before the physiological and biochemical analyses. All physiological and biochemical analyses were performed in triplicates.

### 2.2 Electrolyte leakage determination

Electrolyte or solute leakage was used to evaluate membrane damage when plants were exposed to various environmental stresses (Campos et al., 2003). Electrolyte leakage was performed according to Rolny et al. (2011) using a conductivity meter (HI 98129, Hanna Instruments Ltd.). Briefly, ten freshly cut leaf discs (from a young fully expanded leaf) (0.5 cm in diameter each) were floated in 10 mL of double distilled water (2X dH_2_O) with continuous shaking at 300 rpm in an IKA Vibrax VXR Orbital Shaker (Janke and Kunkel). The electrolyte content in the solution was measured immediately (C_0_), after 3 h (C3) and after 10 min of boiling at 95°C (C_T_). The EL was expressed as a percentage (%) of electrolyte leakage: % EL = 100 x (C_3_‐C_0_)/C_T_.

### 2.3 Malondialdehyde determination

The byproduct of lipid peroxidation, MDA, was determined according to a method modified by Heath and Packer (1968). Frozen leaf tissue (0.3 g) was homogenised to a fine paste in 2.5 mL of 20% (w/v) trichloroacetic acid (TCA) (Sigma‐Aldrich, Germany). The mixture was centrifuged at 3500 *g* at 4°C for 20 min. A 0.5 mL of 20% TCA containing 0.5% (w/v) thiobarbituric acid (TBA) (Sigma‐Aldrich) was added to a tube containing 0.5 mL aliquot and vortexed. The mixture was then incubated for 30 min at 95°C. The tubes were cooled on ice, and the absorbance was measured at 532 and 600 nm (Cary 100 Bio) for the MDA‐TBA product. The MDA content was calculated using an extinction coefficient of 155 mM^−1^ cm^−1^.

### 2.4 Hydrogen peroxide quantification

The hydrogen peroxide (H_2_O_2_) assay was determined according to a modified method described by Velikova et al. (2000). To a 0.3 g frozen leaf tissue, a 2 mL ice cold 0.1% TCA (w/v) was added, homogenised and centrifuged (12000 *g* at 4°C) for 15 min. To a 0.5 mL aliquot, 0.5 mL of 10 mM potassium phosphate buffer (pH 7.0) and 1 mL of 1 M potassium iodide (Merck, South Africa) were added. Absorbance was measured at 390 nm (Cary 100 Bio). The H_2_O_2_ content was calculated from an H_2_O_2_ standard curve (Sigma‐Aldrich) subjected to similar conditions.

### 2.5 Enzyme extraction from leaf tissue

Enzymes were extracted using a method described by Pukacka and Ratajczak (2005). Extraction buffer (3 mL, 50 mM potassium phosphate buffer, pH 7.0) was added to a 0.3 g frozen leaf powder and homogenised to a fine paste. The extraction buffer contained 1 mM ethylenediaminetetraacetic acid (EDTA) (Sigma‐Aldrich), 1 mM ascorbic acid (Sigma‐Aldrich), 2% (w/v) polyvinylpyrrolidone (PVP) (Sigma‐Aldrich) and 0.1% (v/v) Triton X‐100 (BDH Chemicals Ltd.). The homogenate was centrifuged (15000 *g* at 4°C) for 20 min. Protein concentration was determined according to Bradford (1976) using 1.5 mg mL^−1^ gamma‐globulin standard.

### 2.6 Determining the activity of ROS‐eliminating enzymes

The enzyme activity of ascorbate peroxidase (APX, EC 1.11.1.11) was performed according to a method described by Mishra et al. (1993) with modifications. The 1 mL reaction mixture contained 0.55 mL of 50 mM phosphate buffer (pH 7.0), 0.2 mL of 0.1 mM H_2_O_2_, 0.15 mL of 0.5 mM ascorbic acid, 0.05 mL of 0.1 mM EDTA and 0.05 mL enzyme extract. A decrease in absorbance because of ascorbic acid oxidation was measured at 290 nm for 3 min at 20°C against a blank. An extinction coefficient of 2.8 mM^−1^ cm^−1^ was used to calculate APX enzyme activity.

A method described by Zieslin and Ben‐Zaken (1992) was used for the determination of guaiacol peroxidase (GPOX, EC 1.11. 1.7) activity. The reaction mixture contained 50 μL of 0.2 mM H_2_O_2_, 100 μL of 50 mM guaiacol (Sigma‐Aldrich), 0.34 mL double distilled H_2_O, 0.5 mL of 80 mM phosphate buffer (pH 5.5) and 0.01 mL enzyme extract. An increase in absorbance because of tetraguaiacol formation was measured at 470 nm for 3 min at 30°C against a blank. Tetraguaiacol extinction coefficient of 26.6 mM^−1^ cm^−1^ was used to calculate GPOX enzyme activity.

Glutathione reductase (GR, EC 1.8.1.7) activity was determined by following the rate of nicotinamide adenine dinucleotide phosphate (NADPH) (Roche diagnostics GmbH) oxidation at 340 nm for 3 min at 25°C. The assay mixture contained 0.23 mL of 0.2 mM NADPH, 0.23 mL of 0.5 mM oxidized glutathione (GSSG) (Sigma‐Aldrich), 0.03 mL of 2 mM EDTA, 0.47 mL of 100 mM phosphate buffer pH 7.8, and 0.04 mL of enzyme extract. The NADPH extinction coefficient of 6.22 mM^−1^ cm^−1^ was used to calculate glutathione reductase activity.

Superoxide dismutase (SOD, EC 1.15.1.1) activity was performed according to Xu et al. (2008). Briefly, 0.015 mL of enzyme extract was mixed with 1.28 mL of 100 mM phosphate buffer (pH 7.8), 0.04 mL of 55 mM methionine (Merck), 0.15 mL of 0.75 mM nitroblue tetrazolium (NBT) (Sigma‐Aldrich) and 0.03 mL of 0.1 mM riboflavin (Sigma Chemical Co.) in a test tube. The test tubes containing the reaction solution were irradiated under a set of fluorescent light tubes of 40 μmol m^−2^ s^−1^ for 30 min. The absorbance of the irradiated and non‐irradiated solutions was determined at 560 nm. One unit SOD activity was defined as the amount of enzyme that would inhibit 50% NBT photoreduction.

### 2.7 Ascorbic acid quantification

Ascorbic acid (AsA) is an antioxidant and is known to protect cells from oxidative damage under different environmental stresses (Khazaei et al., 2020). Total AsA content was done according to Kampfenkel et al. (1995), and modified by Pukacka and Ratajczak (2005). Briefly, 0.3 g of fine frozen leaf sample was mixed with 2 mL of 6% (w/v) TCA and homogenized until completely thawed and allowed to stand on ice for 15 min. The homogenate was centrifuged at 15600 *g* for 5 min at 4°C. The supernatant was transferred to a new reaction vessel (on ice) and immediately used to determine AsA concentration. In new Eppendorf tubes, 0.2 mL of 0.2 M phosphate buffer (pH 7.4) and 0.1 mL extract were added (the blank contained 0.1 mL of 6% TCA). A 0.1 mL of 10 mM dithiothreitol (DTT) (Sigma‐Aldrich), dissolved in 0.2 M phosphate buffer (pH 7.4), was also added into the tubes followed by the addition of 0.1 mL of 0.5% (w/v) N‐ethylmaleimide (NEM) (Sigma‐Aldrich), 0.5 mL of 10% (w/v) TCA, 0.4 mL of 42% (w/v) ortho‐phosphoric acid (H_3_PO_4_) (Sigma‐Aldrich), 0.4 mL of 4% (w/v) 2,2′‐bipyridyl (Sigma‐Aldrich) (dissolved in 70% (v/v) ethanol), and 0.2 mL of 3% (w/v) iron (III) chloride (FeCl_3_) (Merck). The mixture was incubated at 42°C for 40 min and centrifuged at 1360 *g* for 5 min, and the total AsA content was measured at 525 nm, according to the AsA standard.

### 2.8 Extraction and quantification of lignin and total phenol

It was necessary to elucidate cell wall polysaccharide modifications under DH stress because cell walls act as the first line of defence against different environmental stresses (Le Gall et al., 2015). The alkaline extraction method was performed to extract phenols, lignin, and xylans, according to Gufe et al. (2023), with minor modifications. Soluble sugars were removed from 100 mg dry leaf powder (dried at 60°C for 72 h) with 2 mL of 80% (v/v) ethanol (VWR Chemicals) according to Zhao et al. (2010). This step was repeated three times, the supernatant was discarded. The remaining pellet was then washed twice with 2X distilled water and then suspended in 3 mL of 15% (w/v) sodium hydroxide (NaOH) (BDH Chemicals) and incubated at 60°C for 3 h with agitation every 30 min. After incubation, the mixture was centrifuged at 3000 *g*, the supernatant was taken and neutralised to pH 5.0 with glacial acetic acid. Lignin and phenols were measured immediately from the supernatant. For lignin, the aliquot was diluted 10X with distilled water, and the absorbance was determined at 280 nm. Rutin (Sigma Chemical Co.) was used as the lignin standard. A 3X diluted Folin–Ciocalteu (F–C) reagent (Sigma‐Aldrich) and 35% (w/v) sodium bicarbonate (Sarchem, RSA) were used as detection reagents for total phenolic content. The absorbance was measured at 765 nm and the phenolic content was calculated from a gallic acid (Sigma‐Aldrich) standard.

### 2.9 Hemicellulose and cellulose determination

The xylan from the supernatant (2 mL) was precipitated by adding 6 mL of 95% (v/v) ice‐cold ethanol and incubated overnight at 4°C, according to Gufe et al. (2023). After centrifugation at 3000 *g*, 0.02 mL of the pellet (xylan) was diluted with 2 mL of 2X distilled water. For xylan detection, 0.5 mL of the aliquot was mixed with 0.5 mL of 5% (w/v) phenol (Sigma‐Aldrich) and 1 mL of 95% (v/v) sulphuric acid (H_2_SO_4_) (Merck). The mixture was incubated at 30°C for 5 minutes, and the absorbance was measured at 490 nm. Xylans were determined from a xylose standard (Sigma‐Aldrich).

The remaining pellet in the tubes after the alkaline extraction was used for cellulose determination, according to Palliprath et al. (2020), with minor modifications. The pellet was rinsed 3 times with 2X distilled water, then 1 mL of 73% (v/v) H_2_SO_4_ was added, followed by incubation at 30°C for 60 min. The samples were then diluted to 4% (v/v) H_2_SO_4_ with 2X distilled water and autoclaved at 120°C for 20 min. The phenol and concentrated H_2_SO_4_ method were used for glucose detection, and absorbance was measured at 490 nm. Glucose content was determined using a glucose standard (Sigma‐Aldrich). Holocellulose (%) was calculated by adding cellulose (%) and hemicellulose (%).

### 2.10 Fourier transform infrared spectroscopy (FTIR)

The Fourier transform infrared spectroscopy (FTIR) is an effective, non‐destructive method used to study the chemical composition of cell walls using the function groups (Fadlelmoula et al., 2022). To verify the cell wall alterations of edamame under DH stress, the functional groups in the cell walls of edamame cultivars were analysed with FTIR, according to Trilokesh and Uppuluri (2019). The FTIR of edamame leaf powder, which was dried at 60°C for 72 h, was recorded at room temperature using the Nicolet iS20 Smart iTX FTIR instrument (Thermo Fischer Scientific). All FTIR spectra were collected at a spectrum resolution of 4 cm^−1^, with 32 co‐added scans per sample over the range of 4000 to 800 cm^−1^, this was done in triplicates. The spectrum was used to perform spectra normalisation, baseline corrections, and peak integration. The spectra of edamame samples were presented as absorbance values, and each value represented the means of the three scans.

### 2.11 Statistical analysis

Statistical analysis was done using Genstat Release 22nd edition and Statistica Release 7 software for all biochemical and physiological parameters (except FTIR). The collected data was tested for normality using the Shapiro–Wilk normality test. A split‐split‐plot analysis of variance (ANOVA) was performed based on the split‐split‐plot RCBD to determine the separate and combined effects of temperature, water treatment, and cultivar. The means were separated by Fischer's protected least significant difference (LSD) test at *p* = 0.05, differences or similarities between treatments and cultivars are represented by alphabet letters. Pearson's correlations were performed to determine the relationship between all the physiological and biochemical parameters. Sigma plot version 11 was used to prepare all the figures.

## 3 RESULTS

This study examined the effect of simultaneous drought and heat (DH) stress on reactive oxygen species (ROS), antioxidant responses, and cell wall responses in three edamame varieties. Drought and heat treatments were the positive controls, while well‐watered plants grown at 25°C were the negative controls.

**FIGURE 1:**
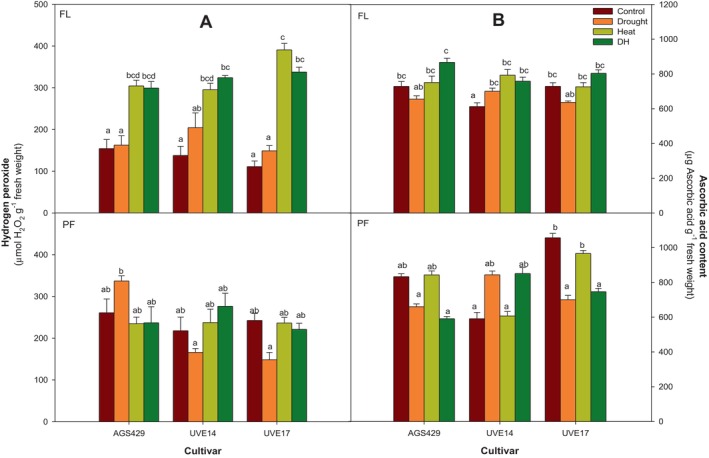
Hydrogen peroxide (A) and ascorbic acid content (B) in three edamame cultivars under drought, heat, combined drought, and heat (DH) stress at flowering (FL) and pod‐filling (PF) growth stages. Alphabets represent similarities or differences in hydrogen peroxide or ascorbic acid content between treatments and cultivars. Values represent means ± SD (*n* = 3). Control = 100% WHC + 25/18°C, Drought = 30% WHC + 25/18°C, Heat = 100% WHC + 35/27°C, DH = 30% WHC + 35/27°C.

Figure 1 shows the levels of hydrogen peroxide (H_2_O_2_) and ascorbic acid (AsA) in three edamame cultivars under DH stress at flowering and pod‐filling stages. These parameters are critical for understanding oxidative stress responses and antioxidative mechanisms in plants, providing insights into how different cultivars cope with combined drought and heat stress. During flowering, all cultivars showed higher H_2_O_2_ levels under combined drought and heat (DH) stress compared to the control, with increases of 48% for AGS429, 58% for UVE14, and 67% for UVE17. The DH stress also caused greater H_2_O_2_ production than drought alone in all cultivars. The H_2_O_2_ levels were similar under heat and DH stress for all cultivars. During the pod‐filling stage, none of the cultivars showed significant changes in H_2_O_2_ production under DH stress compared to the control, drought, or heat stress (Figure 1A).

Although non‐significant, DH treatment in AGS429 increased AsA levels (16% compared to the control and 24% compared to drought treatment). In UVE14, DH stress significantly increased AsA accumulation by 19% compared to the control. There were no significant differences between DH and drought or heat treatments for this cultivar. At the pod‐filling stage, however, there were no significant differences in AsA levels between DH and drought or heat treatments for this cultivar. The DH treatment significantly reduced AsA levels only in UVE17 compared to the control (35%) and heat (18%) treatments (Figure 1B).

**FIGURE 2:**
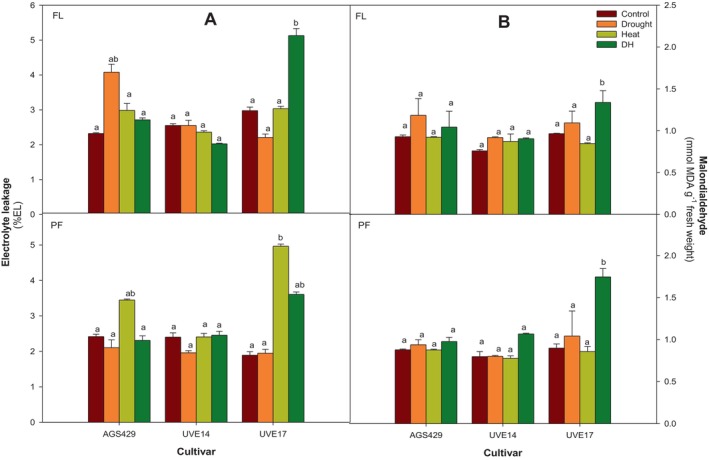
Electrolyte leakage (A) and (B) malondialdehyde contents in three edamame cultivars under combined drought and heat (DH) stress at the flowering (FL) and pod‐filling (PF) growth stages. Alphabets represent similarities or differences in electrolyte leakage or malondialdehyde content between treatments and cultivars. Values represent means ± SD (n = 3). Control = 100% WHC + 25/18°C, Drought = 30% WHC + 25/18°C, Heat = 100% WHC + 35/27°C, DH = 30% WHC + 35/27°C.

Figure 2 shows the electrolyte leakage (EL) and malondialdehyde (MDA) levels in the three edamame cultivars following DH treatment. Measuring these important indicators of cell membrane integrity and lipid peroxidation helps to assess the extent of cellular damage under combined drought and heat stress in these cultivars. UVE17 displayed a significantly higher EL under DH treatment than the negative control (42% increase at the flowering stage). Though insignificant, UVE17 plants showed an increase in EL under DH treatment compared to the control and drought alone at pod‐filling. No significant differences were observed between DH treatment and controls for UVE14 at either growth stage. AGS429 showed no difference in EL under DH stress compared to the control, drought and heat treatments during flowering and pod‐filling stages (Figure 2A). There were no significant differences between DH treatment and controls for UVE14 and AGS429 at either growth stage in terms of MDA accumulation. However, UVE17 had the highest MDA levels under DH stress at flowering and pod‐filling stages compared to the negative and positive controls (Figure 2B).

**FIGURE 3:**
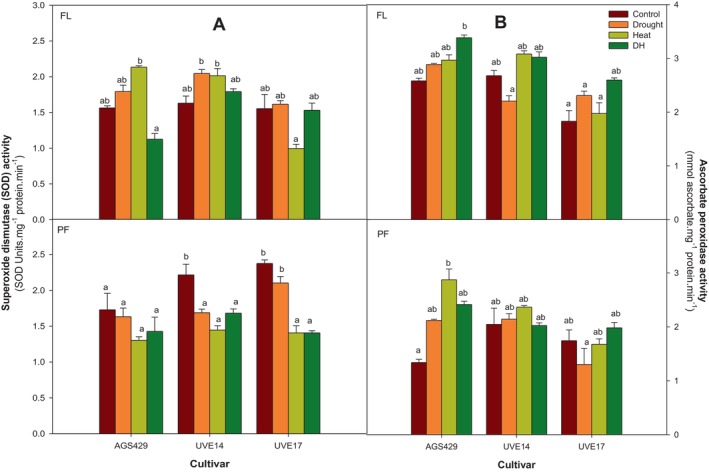
Superoxide dismutase (A) and ascorbate peroxidase (B) enzyme activities of three edamame cultivars under drought, heat, combined drought, and heat (DH) stress at flowering (FL) and pod‐filling (PF) growth stages. Alphabets represent similarities or differences in the antioxidative enzyme activities between treatments and cultivars. Values represent means ± SD (n = 3). Control = 100% WHC + 25/18°C, Drought = 30% WHC + 25/18°C, Heat = 100% WHC + 35/27°C, DH = 30% WHC + 35/27°C.

This section presents the results of the antioxidative enzyme activities, which play crucial roles in mitigating oxidative stress and protecting plants from damage caused by environmental stress. Figure 3 represents superoxide dismutase (SOD) and ascorbate peroxidase (APX) activities in three edamame cultivars under DH stress at two growth stages. At the flowering stage, compared to the control, DH treatment did not significantly affect SOD activity in all cultivars. At the pod‐filling, DH treatment in UVE14 substantially reduced the SOD activity by 24% when compared to the control. In UVE17, DH stress reduced SOD activity by 41% and 38% compared to the control and drought treatments, respectively. Notably, the SOD activity in AGS429 was unaffected under DH treatment. There were no differences between DH treatment and drought or heat treatment in AGS429 and UVE14 (Figure 3A). Although the increase was not statistically significant, DH treatment raised APX activity in AGS429 (23%) and UVE17 (30%) compared to the control at the flowering stage. Additionally, in AGS429, APX activity under DH stress was higher than under drought (15%) and heat (12%) treatments, though the differences were not significant. Similarly, DH treatment induced a substantial increase in APX activity in UVE17 in relation to the drought (12%) or heat (24%) treatments. At pod‐filling, APX activities in UVE14 and UVE17 were not influenced by DH compared to other treatments. While the change in APX activity under DH stress in AGS429 did not reach statistical significance at pod‐filling, the 44% increase compared to the control remains noteworthy (Figure 3B).

**FIGURE 4:**
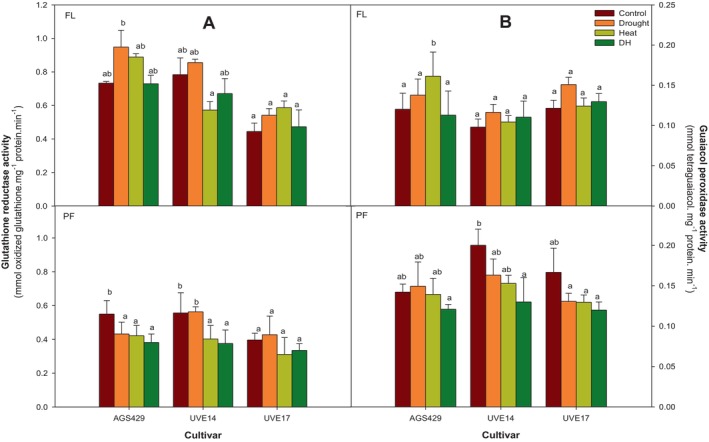
Glutathione reductase (A) and guaiacol peroxidase (B) enzyme activities of three edamame cultivars under drought, heat, combined drought, and heat (DH) stress at flowering (FL) and pod‐filling (PF) growth stages. Alphabets represent similarities or differences in the antioxidative enzyme activities between treatments and cultivars. Values represent means ± SD (n = 3). Control = 100% WHC + 25/18°C, Drought = 30% WHC + 25/18°C, Heat = 100% WHC + 35/27 C, DH = 30% WHC + 35/27°C.

Figure 4 represents the glutathione reductase (GR) and guaiacol peroxidase (GPOX) in three edamame cultivars under DH stress at two growth stages. Together with APX and SOD, these enzymes perform antioxidative roles in plants, especially under stress. At the flowering stage, GR activity in AGS429 was similar between the control and DH‐treated plants. However, drought and heat treatments increased GR activity, although not significantly (Figure 4A). Additionally, there were no significant differences in GR activity between UVE17 and UVE14 for the control, drought, and DH treatments. At pod‐filling, the DH stress significantly reduced GR activity levels by 31% compared to the control in AGS429; however, there were no significant differences between drought, heat and DH treatments. The DH treatment in UVE14 resulted in a 33% decrease in GR activity compared to the control and drought treatments, but for UVE17, GR activity was not significantly different between controls and DH treatment (Figure 4A). Figure 4B shows that GPOX activity levels were not significantly different between all cultivars exposed to control, drought, heat and DH stress conditions; the only exception was observed in AGS429, where GPOX activity level increased by 30% under heat stress conditions. Although a 15% GPOX activity reduction was observed in AGS429 at pod‐filling under DH compared to the control, the highest reduction in the enzyme activity was observed in UVE14 (35%) and UVE17 (29%) compared to controls (Figure 4B).

**FIGURE 5:**
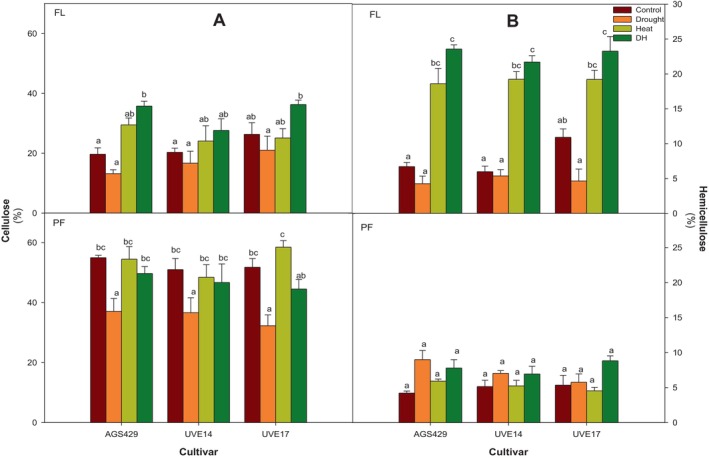
Cellulose (A) and hemicellulose (B) content of three edamame cultivars under drought, heat, combined drought, and heat (DH) stress at flowering (FL) and pod‐filling (PF) growth stages. Alphabets represent similarities or differences in cellulose or hemicellulose content between treatments and cultivars. Values represent means ± SD (n = 3). Control = 100% WHC + 25/18°C, Drought = 30% WHC + 25/18°C, Heat = 100% WHC + 35/27°C, DH = 30% WHC + 35/27°C.

Figure 5 represents the cellulose and hemicellulose content in three edamame cultivars under DH stress at two growth stages. These components help maintain cell wall strength, which contributes to plant survival under environmental stress, providing insight into the resilience of the cultivars. The DH stress significantly increased cellulose content by 45% in AGS429 compared to the control at the flowing stage. In addition, drought had the lowest cellulose contents for all the cultivars; however, AGS429 and UVE14, under heat stress, had the second highest cellulose content (Figure 5A). At pod‐filling, DH did not affect cellulose content in AGS429 and UVE14 compared to the control and heat treatments, but in UVE17, there was a 14% reduction in cellulose content compared to the control and a 24% reduction compared to heat alone. However, compared to drought alone, DH resulted in higher cellulose content in AGS429 (25% increase), UVE14 (22% increase) and UVE17 (27% increase) (Figure 5A). The DH resulted in the highest hemicellulose content in AGS429, UVE14, and UVE17 (71, 72, and 53%, respectively) compared to the control. For all cultivars, DH treatment did not differ significantly from heat treatment. However, a significant increase was observed under DH stress compared to drought treatment in all cultivars. At pod‐filling, there was an insignificant accumulation of hemicellulose under DH stress in all cultivars compared to the controls (Figure 5B).

**FIGURE 6:**
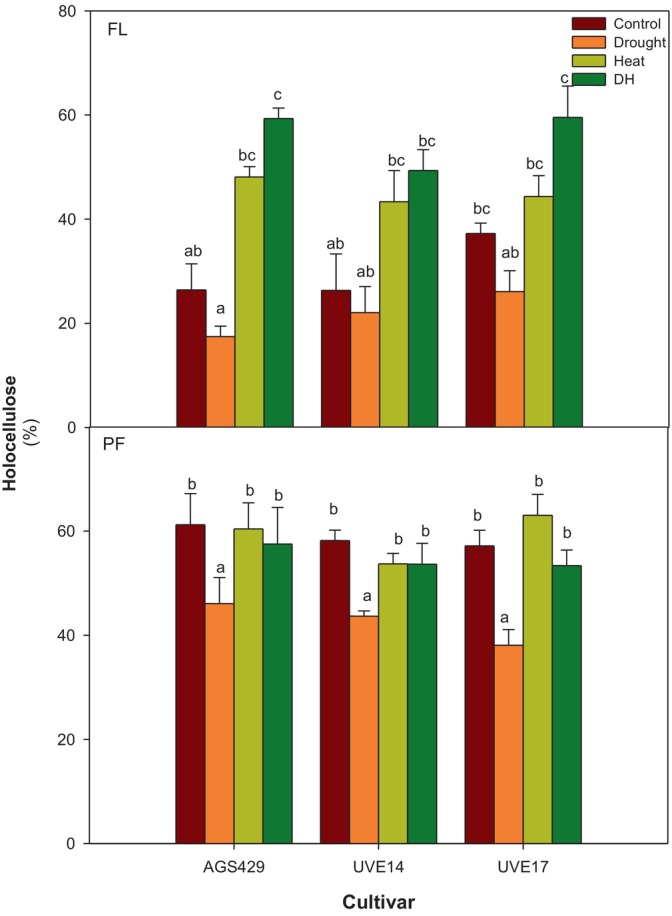
Holocellulose content of three edamame cultivars under drought, heat, combined drought, and heat (DH) stress at flowering (FL) and pod‐filling (PF) growth stages. Alphabets represent similarities or differences in holocellulose content between treatments and cultivars. Values represent means ± SD (n = 3). Control = 100% WHC + 25/18°C, Drought = 30% WHC + 25/18°C, Heat = 100% WHC + 35/27°C, DH = 30% WHC + 35/27°C.

The holocellulose content corroborates the sum of cellulose and hemicellulose, resulting in three edamame cultivars under DH stress (Figure 6). At flowering, the DH stress led to the accumulation of holocellulose in all cultivars compared to the control and drought treatments. The holocellulose accumulation significantly increased by 55% in AGS429 compared to the control. DH treatment was not significantly different from heat treatment for all cultivars. However, it led to a significant increase in holocellulose compared to drought‐stressed cultivars. At pod‐filling, the holocellulose content under DH stress was not significantly different to the control and heat treatment but significantly increased (20, 19, and 29%) in AGS429, UVE14, and UVE17, respectively, compared to drought treatment.

**FIGURE 7:**
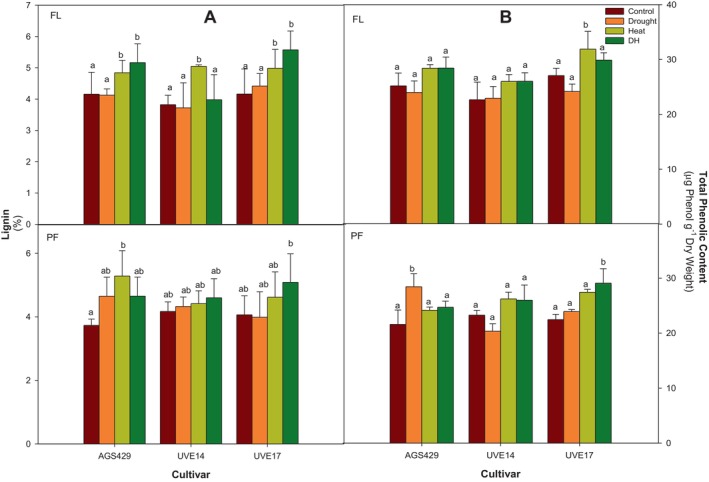
Lignin (A) and total phenolic (B) content of three edamame cultivars under drought, heat, combined drought, and heat (DH) stress at flowering (FL) and pod‐filling (PF) growth stages. Alphabets represent similarities or differences in lignin or total phenolic content between treatments and cultivars. Values represent means ± SD (n = 3). Control = 100% WHC + 25/18°C, Drought = 30% WHC + 25/18°C, Heat = 100% WHC + 35/27°C, DH = 30% WHC + 35/27°C.

Figure 7 represents the lignin and phenolic content in three edamame cultivars under DH stress at two growth stages. These compounds play a vital role in strengthening the plant cell wall and provide protection against oxidative damage. At the flowering stage, DH treatment significantly increased lignin accumulation in AGS429 (19%) and UVE17 (25%) compared to the control (Figure 7A). At pod‐filling stage, a significant increase in lignin was observed only in UVE17 (19%) compared to the control. At the flowering stage, the phenolic content for all cultivars under DH stress was not different from that of the controls. However, the DH stress at pod‐filling led to a 23% accumulation of phenolic compounds compared to the control in UVE17. In AGS429, phenolic content was 13% lower than that of drought treatment but not different from that of control and heat treatment (Figure 7B).

**FIGURE 8:**
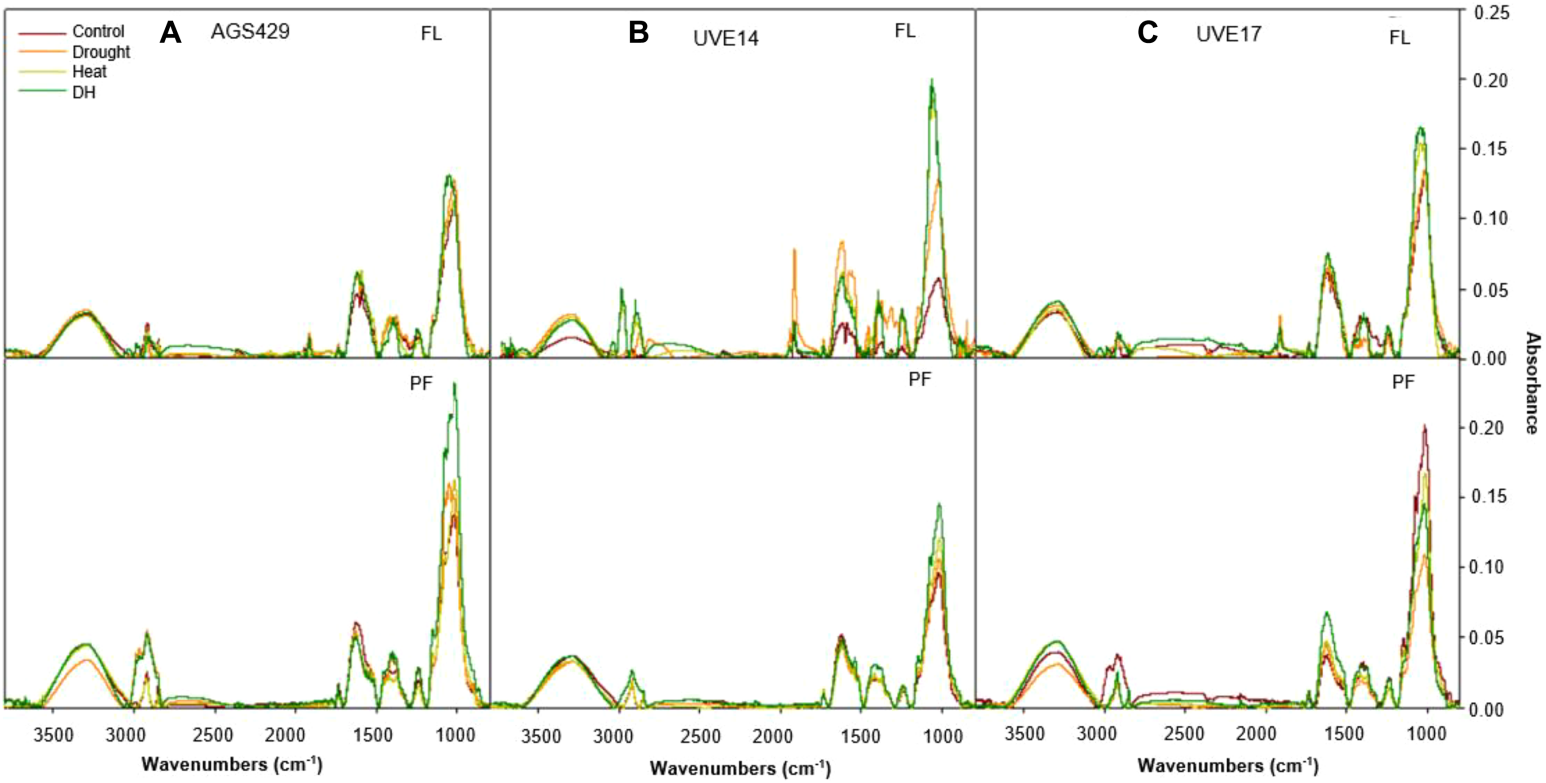
Fourier transform infrared (FTIR) spectra of three edamame cultivars under drought, heat, combined drought, and heat (DH) stress at flowering (FL) and pod‐filling (PF) growth stages. Spectrum for cell wall composition was performed in the region between 4000–800 cm^−1^ at 4 cm^−1^ spectral resolution. Each sample was scanned 32X and collected in absorbance mode. Control = 100% WHC + 25/18°C, Drought = 30% WHC + 25/18°C, Heat = 100% WHC + 35/27°C, DH = 30% WHC + 35/27°C. drought stress, UVE14 = low yielding with low yield reduction under drought stress, UVE17 = low yielding with high yield reduction under drought stress.

Figure 8 represents the Fourier transform infrared (FTIR) spectra in three edamame cultivars under DH stress at two growth stages. This analysis complements the measurements of cellulose and hemicellulose content, providing additional insights into the composition and structural changes and helping to assess the overall resilience of edamame under DH stress. At the flowering stage in AGS429, DH stress increased holocellulose (1200–1000 cm^−1^) compared to the control and heat treatment (Figure 8A). In relation to the control, the unconjugated hemicellulose (1733 cm^−1^) was also increased in AGS429 at flowering. At pod‐filling, the crystalline cellulose (3500–3000 cm^−1^) under DH stress was not different from the control and heat treatment, and the unconjugated hemicellulose was low in relation to the control. The holocellulose content was elevated under DH stress in AGS429 compared to all the controls (Figure 8A). The lignin in AGS429 at 1699–1430 cm^−1^ was slightly high under DH stress compared to control and other treatments. In UVE14, DH stress resulted in increased crystalline cellulose compared to the control, which was slightly lower than separate treatments of drought and heat (Figure 8B). The unconjugated hemicellulose was higher than the control under DH stress but somewhat lower than heat and drought treatments. The holocellulose was elevated compared to control and drought. At pod‐filling, the crystalline cellulose under DH was not different compared to control and substantially elevated compared to drought and heat. The lignin and holocellulose were elevated compared to all controls. At the flowering stage, UVE17 had most of its cell wall polysaccharides elevated under DH stress compared to all the control, drought, and heat, as shown by the highest crystalline cellulose content, unconjugated hemicellulose, and holocellulose (Figure 8C). However, at pod‐filling, the holocellulose was remarkably low under DH stress compared to the control. The lignin content at both flowering and pod‐filling growth stages was not different to the control in UVE17. Tables 1, 2, and 3 represent the correlations between the different biochemical and cell wall responses in three edamame cultivars under DH stress at two growth stages. These analyses are crucial for understanding how the biochemical processes are interconnected in response to DH stress, providing insights that can inform strategies to enhance resilience in edamame. In all cultivars at the flowering stage, holocellulose strongly correlated with cellulose (*p* ≤ 0.01). Cellulose and holocellulose strongly negatively correlated in UVE17 (Table 3) at pod‐filling (*p* ≤ 0.01), but UVE14 showed a strong positive correlation between cellulose and holocellulose at pod‐filling (*p* ≤ 0.01). In AGS429 (Table 1), cellulose strongly positively correlated with TP (*p* ≤ 0.01) and lignin (*p* ≤ 0.001) at pod‐filling. Furthermore, in this cultivar, H_2_O_2_ positively correlated with APX (*p* ≤ 0.01) at flowering and cellulose at both growth stages (*p* ≤ 0.001). At flowering, APX positively correlated with AsA in AGS429 (*p* ≤ 0.001), while MDA had a strong negative association with holocellulose and AsA (*p* ≤ 0.01) at pod‐filling. In UVE14 (Table 2), there was a strong positive correlation between H_2_O_2_ and cellulose at pod‐filling (*p* ≤ 0.001), and MDA negatively correlated with AsA at flowering (*p* ≤ 0.001). At both growth stages, APX positively correlated with AsA in UVE14. In UVE17 (Table 3), H_2_O_2_ negatively correlated with holocellulose (*p* ≤ 0.001), total phenolic content (*p* ≤ 0.01), and cellulose (*p* ≤ 0.01) at pod‐filling. Hydrogen peroxide positively correlated with EL (*p* ≤ 0.01) at pod‐filling. At pod‐filling, MDA negatively correlated with holocellulose (*p* ≤ 0.001), and positively correlated with EL at both growth stages. Electrolyte leakage negatively correlated with holocellulose and cellulose in UVE17 at pod‐filling (*p* ≤ 0.01).

**TABLE 1:** The relationship between various physio‐biochemical traits at the flowering and pod‐filling stage in AGS429 under combined drought and heat (30% WHC, 35/28°C) stress.

AGS429	Parameter	H_2_O_2_	MDA	SOD	GPOX	GR	APX	Holcel	Lignin	Hemcel	TP	Cellulose	AsA	EL
Flowering	**H**_**2**_ **O**_**2**_	‐												
	**MDA**	−0.69	‐											
	**SOD**	0.63	−0.96**	‐										
	**GPOX**	−0.91**	0.71	−0.65	‐									
	**GR**	−0.73	0.02	0.07	0.71	‐								
	**APX**	0.75*	0.52	−0.59	−0.23	−0.81*	‐							
	**Holcel**	0.95**	−0.66	0.60	−0.91**	−0.38	0.29	‐						
	**Lignin**	0.72	0.01	−0.09	−0.71	−0.93**	0.86*	0.74	‐					
	**Hemcel**	0.01	0.71	−0.77	0.04	−0.73	0.97**	0.05	0.73	‐				
	**TP**	0.98**	−0.71	0.64	−0.92**	−0.80*	0.23	0.98**	0.70	−0.01	‐			
	**Cellulose**	0.98**	−0.81*	0.76	−0.90**	−0.61	0.08	0.88*	0.67	−0.16	0.97**	‐		
	**AsA**	0.03	0.70	−0.76	0.04	−0.74	0.97**	0.07	0.74	0.97**	0.01	−0.15	‐	
	**EL**	0.34	0.37	0.73	−0.04	0.55	0.67	−0.17	0.32	0.33	0.13	0.72	0.22	‐
Pod‐filling	**H**_**2**_ **O**_**2**_	‐												
	**MDA**	−0.19	‐											
	**SOD**	0.44	0.80*	‐										
	**GPOX**	0.70	−0.83*	−0.34	‐									
	**GR**	0.04	−0.99**	−0.88*	0.74	‐								
	**APX**	0.43	0.97**	0.63	−0.95**	−0.92**	‐							
	**Holcel**	0.66	−0.86*	−0.39	0.94**	0.80*	−0.66	‐						
	**Lignin**	0.93**	−0.10	0.51	0.64	−0.04	−0.35	0.59	‐					
	**Hemcel**	0.06	−0.99**	−0.87*	0.80*	0.98**	−0.93**	0.80*	−0.02	‐				
	**TP**	0.58	0.69	0.99**	−0.18	−0.80*	0.49	−0.24	0.64	−0.80*	‐			
	**Cellulose**	0.97**	0.04	0.63	0.52	−0.18	−0.22	0.47	0.99**	−0.16	0.80*	‐		
	**AsA**	0.99**	−0.80*	0.34	0.80*	0.14	−0.52	0.73	0.98**	0.16	0.49	0.95**	‐	
	**EL**	0.56	−0.68	0.09	0.16	0.33	0.22	−0.44	0.31	0.08	0.56	0.68	−0.35	‐

Values represent the coefficient of determination (R^2^) at * *p* ≤ 0.01, ** *p* ≤ 0.001. APX = Ascorbate peroxidase, AsA = Ascorbic acid, EL = Electrolyte leakage, GPOX = Guaiacol peroxidase, GR = Glutathione reductase, H_2_O_2_ = Hydrogen peroxide, Hemcel = Hemicellulose, MDA = Malondialdehyde, SOD = Superoxide dismutase, Holcel = Holocellulose, TP = Total phenols.

**TABLE 2:** The relationship between various physio‐biochemical traits at flowering and pod‐filling stage in UVE14 under combined drought and heat (30% WHC, 35°C) stress.

UVE14	Parameter	H_2_O_2_	MDA	SOD	GPOX	GR	APX	Holcel	Lignin	Hemcel	TP	Cellulose	AsA	EL	
Flowering	**H**_**2**_ **O**_**2**_	‐												
	**MDA**	−0.04	‐											
	**SOD**	0.97**	0.20	‐										
	**GPOX**	−0.37	0.94**	−0.14	‐									
	**GR**	0.18	0.98**	0.41	0.85*	‐								
	**APX**	−0.19	−0.97**	−0.42	−0.84*	−0.87*	‐							
	**Holcel**	−0.51	−0.84*	−0.70	−0.61	−0.90**	0.91**	‐						
	**Lignin**	0.73	−0.71	0.55	−0.90**	−0.50	0.52	0.21	‐					
	**Hemcel**	0.26	−0.97**	0.02	−0.99**	−0.90**	0.93**	0.70	0.85*	‐				
	**TP**	0.83*	0.52	0.94**	0.20	0.70	−0.74	−0.90**	0.24	−0.31	‐			
	**Cellulose**	−0.98**	0.10	−0.95**	0.43	−0.10	0.14	0.45	−0.80*	−0.32	−0.80*	‐		
	**AsA**	0.26	−0.98**	0.02	−0.99**	−0.90**	0.91**	0.80*	0.85*	0.98**	−0.31	−0.32	‐	
Pod‐filling	**EL**	0.56	0.43	0.08	−0.52	0.13	−0.43	0.32	−0.34	0.05	−0.65	0.43	−0.11	‐
	**H**_**2**_ **O**_**2**_	‐												
	**MDA**	0.29	‐											
	**SOD**	−0.57	0.63	‐										
	**GPOX**	0.80*	−0.42	−0.97**	‐									
	**GR**	0.98**	0.19	−0.64	0.81*	‐								
	**APX**	−0.98**	−0.21	0.63	−0.86*	−0.91**	‐							
	**Holcel**	0.97**	0.05	−0.80*	0.94**	0.93**	−0.90**	‐						
	**Lignin**	−0.94**	0.04	0.81*	−0.91**	−0.91**	0.93**	−0.98**	‐					
	**Hemcel**	−0.44	−0.99**	−0.49	0.32	−0.41	0.42	−0.21	0.12	‐				
	**TP**	−0.69	0.49	0.99**	−0.90**	−0.82*	0.71	−0.85*	0.89*	−0.34	‐			
	**Cellulose**	0.91**	0.66	−0.17	0.44	0.93**	−0.94**	0.80*	−0.72	−0.80*	−0.33	‐		
	**AsA**	−0.73	0.45	0.98**	−0.90**	−0.82*	0.81*	−0.87*	0.91**	−0.29	0.97**	−0.38	‐	
	**EL**	−0.57	0.19	0.23	−0.32	0.71	0.22	0.32	−0.09	0.43	0.13	−0.35	−0.43	‐

Values represent the coefficient of determination (R^2^) at *p ≤ 0.01, ** *p* ≤ 0.001. APX = Ascorbate peroxidase, AsA = Ascorbic acid, EL = Electrolyte leakage, GPOX = Guaiacol peroxidase, GR = Glutathione reductase, H_2_O_2_ = Hydrogen peroxide, Hemcel = Hemicellulose, MDA = Malondialdehyde, SOD = Superoxide dismutase, Holcel = Holocellulose, TP = Total phenols.

**TABLE 3:** The relationship between various physio‐biochemical traits at flowering and pod‐filling stage in UVE17 under combined drought and heat (30% WHC, 35°C) stress.

UVE17	Parameter	H_2_O_2_	MDA	SOD	GPOX	GR	APX	Holcel	Lignin	Hemcel	TP	Cellulose	AsA	EL
Flowering	**H**_**2**_ **O**_**2**_	‐												
	**MDA**	0.85*	‐											
	**SOD**	0.55	−0.91**	‐										
	**GPOX**	−0.59	0.08	0.35	‐									
	**GR**	−0.93**	0.92**	−0.67	0.46	‐								
	**APX**	0.20	−0.69	0.93**	0.67	−0.35	‐							
	**Holcel**	0.92**	−0.73	0.83*	−0.23	−0.97**	0.57	‐						
	**Lignin**	0.86*	−0.86*	0.56	−0.57	−0.99**	0.22	0.93**	‐					
	**Hemcel**	0.91**	−0.56	0.15	−0.87*	−0.84*	−0.22	0.68	0.90**	‐				
	**TP**	0.25	0.30	−0.68	−0.93**	−0.10	−0.90*	−0.15	0.22	0.62	‐			
	**Cellulose**	0.84**	−0.93**	0.86*	−0.17	−0.95**	0.62	0.82*	0.90**	0.63	−0.21	‐		
	**AsA**	0.86*	−0.46	0.04	−0.92**	−0.77	−0.32	0.59	0.85*	0.99**	0.70	0.54	‐	
	**EL**	0.81*	0.88*	−0.21	−0.64	−0.66	0.55	0.73	0.71	0.89	0.45	0.67	0.73	‐
	**H**_**2**_ **O**_**2**_	‐												
	**MDA**	0.13	‐											
	**SOD**	−0.25	−0.91**	‐										
	**GPOX**	0.64	−0.68	0.58	‐									
Pod‐filling	**GR**	−0.93**	0.24	−0.11	−0.87*	‐								
	**APX**	0.98**	−0.06	−0.07	0.77	−0.98**	‐							
	**Holcel**	−0.90**	−0.85*	0.65	−0.23	0.68	−0.80*	‐						
	**Lignin**	0.89*	0.23	−0.36	0.55	−0.89*	0.96**	−0.94**	‐					
	**Hemcel**	−0.94**	−0.45	0.56	−0.34	0.76	−0.87*	0.99**	−0.97**	‐				
	**TP**	−0.83*	−0.20	0.33	−0.58	0.90**	−0.97**	0.93**	−0.88*	0.97**	‐			
	**Cellulose**	−0.80*	−0.70	0.76	−0.04	0.53	−0.67	−0.80*	−0.86*	0.95**	0.80*	‐		
	**AsA**	0.82*	0.23	−0.36	0.55	−0.89*	0.96**	−0.94**	0.98**	−0.97**	−0.96**	−0.86*	‐	
	**EL**	0.80*	0.92**	−0.74	−0.74	−0.78	−0.32	−0.81*	0.73	−0.81*	0.38	−0.88*	−0.54	‐

Values represent the coefficient of determination (R^2^) at *p ≤ 0.01, **p ≤ 0.001. APX = Ascorbate peroxidase, AsA = Ascorbic acid, EL = Electrolyte leakage, GPOX = Guaiacol peroxidase, GR = Glutathione reductase, H_2_O_2_ = Hydrogen peroxide, Hemcel = Hemicellulose, MDA = malondialdehyde, SOD = Superoxide dismutase, Holcel = Holocellulose, TP = Total phenols.

## 4 DISCUSSION

The physiological and biochemical responses to drought and heat stress are often studied separately. However, these stresses frequently occur together in nature, eliciting distinct plant responses under combined drought and heat (DH) stress compared to individual stress conditions (Cohen et al., 2021). The higher accumulation of the reactive oxygen species (ROS) under DH stress can damage cell membranes and cause plant cells to leak out electrolytes such as potassium ions, thus compromising the cell membrane's fluidity and integrity, making membrane‐bound organelles such as chloroplast and mitochondria dysfunctional (Hatsugai & Katagiri, 2018; Sharma et al., 2023). Hydrogen peroxide (H_2_O_2_) and lipid peroxidation accumulation are classic indicators that plants are under stress (Haddidi et al., 2020). Lipid peroxidation is measured through malondialdehyde (MDA) production, accumulating in plants under stress. Excessive production of H_2_O_2_ in all edamame cultivars at the flowering stage under heat treatment and DH stress indicates that they responded to stress. However, without effective ROS disposal mechanisms, these cultivars may risk generating highly reactive and membrane‐damaging hydroxyl radicals (•OH) (Sainz et al., 2010). In AGS429 and UVE14, ascorbic acid (AsA, an antioxidant) positively correlated with APX at the flowering and pod‐filling stage. This indicates that these two antioxidative systems are co‐dependent for neutralising the ROS under DH stress. The drought‐susceptible cultivar UVE17 did not accumulate AsA, resulting in high MDA and electrolyte leakage at the flowering and pod‐filling stages. These findings indicate potential phospholipid peroxidation and membrane damage in UVE17 due to DH stress (Hussain et al., 2019; Xiao et al., 2021). A strong positive correlation between MDA and EL at both growth stages and a positive correlation between H₂O₂ and EL at the pod‐filling stage in UVE17 shows that DH stress reduces membrane integrity in this cultivar. Other studies showed the importance of AsA in alleviating oxidative stress or quenching the ROS in wheat, strawberry and tomato plants under drought stress or heat stress (Ergin et al., 2014; Kumar et al., 2014; Alayafi, 2020; Xiao et al., 2021).

Superoxide dismutase (SOD) catalyses the dismutation of superoxide anion radical (O_2_^•−^) to H_2_O_2_, which is then reduced by APX or GPOX to H_2_O and monodehydroascorbate (MDHA) by using AsA as an electron donor (Balfagón et al., 2018). Sainz et al. (2010) reported photosystem II (PSII) damage caused by chloroplast Cu/Zn‐SOD degradation under heat stress in DH‐tolerant *Lotus japonicus* plants. While SOD was not affected at pod‐filling stage in AGS429 under DH stress, UVE14 and UVE17 showed a substantial reduction, suggesting poor O_2_^•−^ quenching mechanism under DH stress compared to AGS429. Notably, the accumulation of AsA in UVE14 at the flowering stage, along with elevated APX enzyme activity under DH stress, contributes to reduced H₂O₂ levels, thereby preventing lipid peroxidation (MDA) and minimizing electrolyte leakage. At pod‐filling, strong positive correlations between APX and H_2_O_2_ at the flowering stage indicate that the maintenance of APX activity in AGS429 could serve as a DH tolerance mechanism to prevent the spontaneous formation of •OH. Similarly, increased APX activity in citrus genotypes was one of the tolerant mechanisms against combined drought and heat stress (Balfagón et al., 2018).

Guaiacol peroxidase (GPOX) oxidises guaiacol to form tetraguaiacol in the expense of H_2_O_2_. This enzyme is involved in several biochemical processes, including lignin biosynthesis (Gill & Tuteja, 2010; Radulescu et al., 2019; Uarrota et al., 2016). However, its role in edamame under combined DH stress remains unknown. At flowering, there was no difference in GPOX activity in all cultivars under DH and the control or drought treatment, except that AGS429 showed low GPOX activity compared to the heat treatment. All cultivars displayed a low GPOX activity at pod‐filling compared to the control. Some studies have reported an enhanced GPOX activity under single stresses of drought or heat in spinach, soybean, and wheat (Gupta et al., 2013; Moloi et al., 2016; Liatile et al., 2022). However, in the present study, the reduction in GPOX activity in all cultivars at pod‐filling suggests that the enzyme might not be directly involved in H_2_O_2_ elimination under DH stress in the three edamame cultivars. Moreover, Gill and Tuteja (2010) argued that GPOX activity differs substantially depending on plant genotype and stress conditions.

Glutathione reductase (GR) is an antioxidant enzyme that maintains the reduced glutathione (GSH) pool at the expense of NADPH. Glutathione should be in the reduced (GSH) form in plant cells for antioxidative and metabolic regulatory processes since a high ratio of the oxidized (GSSG) form indicates oxidative stress (Gill & Tuteja, 2010; Kwon et al., 2019). The GR activity in the present study did not have any notable changes under DH stress in all cultivars at the flowering stage compared to the controls, though in AGS429 the activity was lower than in drought treatment. However, at pod‐filling, the high‐yielding, drought‐tolerant cultivar AGS429 and the drought‐tolerant UVE14 had reduced GR activity under DH, while the drought‐susceptible UVE17 showed no difference. Since GR depends on NADPH to reduce the GSSG disulphide bond, the low GR activity in AGS429 and UVE14 could be due to high NADPH demand for seed filling and carbohydrates synthesis for cell wall re‐enforcement at pod‐filling, leading to low NADPH available for GR reaction. However, AGS429 seemed to have employed the AsA and APX antioxidative strategy, UVE14 used the AsA mechanism rather than GPOX and GR, while UVE17 lacked a specific antioxidative stress mechanism and resulted in high electrolyte leakage and lipid peroxidation at both growth stages under DH stress. Several studies have reported the involvement of GR in alleviating oxidative stress by maintaining a higher GSH: GSSG ratio in tomato, citrus, and turf grass under single and combined drought and heat stress (Jiang & Huang, 2001; Zandalinas et al., 2017; Raja et al., 2020).

Cell wall restructuring is an important physiological response of plants exposed to drought (Al‐Yasi et al., 2020) or heat stress (Lima et al., 2013). Whether drought or heat stress is applied individually or in combination, the stresses cause cell wall stiffening or loosening, which may result in plant resistance or susceptibility. Cellulose (the main load‐bearing polymer of the cell wall) is bio‐synthesised in the plasma membrane by the membrane‐localised cellulose synthase complexes (CSCs) (Lei et al., 2015). Cellulose synthesis can be compromised under DH stress due to oxidative stress‐linked leakage, dissociation, endocytosis, and recycling of plasma membrane materials, including CSCs (Lei et al., 2015; Kesten et al., 2017). In AGS429 (flowering) and UVE14 (flowering and pod‐filling), the strong positive correlation between APX and holocellulose suggests that membrane damage prevention in the two cultivars improved the holocellulose content. Though all cultivars accumulated cellulose and hemicellulose (with AGS429 having the most significant increase) for cell wall re‐enforcement at the flowering stage under DH, there was a strong negative correlation at pod‐filling between holocellulose (cellulose and hemicellulose) and MDA, suggesting that high lipid peroxidation resulted in poor cell wall carbohydrates synthesis in UVE17. This observation is also confirmed by the reduced cellulose (which strongly negatively correlates with holocellulose at pod‐filling stage in UVE17) content in UVE17 compared to the control and heat treatment at pod‐filling. Cellulose fibers form strong hydrogen bond complexes with hemicellulose to strengthen cell walls under environmental stress (Lima et al., 2013). Therefore, cellulose reduction in UVE17 due to DH stress could result in weak cell walls, making the cultivar susceptible to DH stress. Accordingly, Hu et al. (2022) also observed a substantial reduction in cellulose content and quality in cotton plants under DH stress.

The cell wall comprises cellulose, hemicellulose, phenolics and lignin. Reactive oxygen species also regulate plant cell walls during drought or heat stress. For example, high H_2_O_2_ concentration oxidises cell wall polysaccharides or reacts with the lignin phenolic units, forming auto‐cross‐linking between phenols, making cell walls stiff (Huang et al., 2019; Mnich et al., 2020; Martin et al., 2021; Mafa et al., 2022). Interestingly, the findings of this study show that the H_2_O_2_ accumulated in AGS429, UVE14 and UVE17 at the flowering stage resulted in cell wall holocellulose deposition and lignification during drought, heat or DH stress. Additionally, a strong positive correlation was observed between AsA and total phenolic content in UVE14 at pod‐filling, suggesting that AsA (which has higher oxidation potential than phenols) regenerated and prevented the loss of phenols (Poletto et al., 2021), suggesting enhanced ROS‐dependent oxidative polymerisation in UVE14 under DH stress. The drought‐tolerant AGS429 and susceptible cultivar UVE17 showed increased lignin content under DH stress compared to the controls. Accordingly, Hlahla et al. (2022) observed lignin and phenolics accumulating in six edamame cultivars irrespective of stress tolerance or susceptibility under drought conditions. Other studies also demonstrated that lignin and phenolics accumulated under drought and DH stress in tea plants and desert grass (*Artemisia sieberi alba*). However, it is essential to note that lignification is part of the basal defence under drought stress by strengthening the cell wall through cross‐linking and preventing water loss (Mnich et al., 2020, Hlahla et al., 2022). Likewise, under DH stress, lignin and phenolics provided defence in the three edamame cultivars.

The Fourier transform infrared spectroscopy was used to validate the cell wall modification, which was demonstrated through compositional analysis. The FTIR results confirmed that AGS429 and UVE14 had accumulated holocellulolytic content at both growth stages, as shown by a higher peak representing the C – O, and β‐glycosidic bond stretching at 1000–1200 cm^−1^ (Javier‐Astete et al., 2021; Mafa et al., 2022). The increase in holocellulose, crystalline/amorphous cellulose (C – H stretching, 3000–3500 cm^−1^) (Mohotloane et al., 2024) and lignin content (C = O and aromatic skeletal vibration, 1430–1699 cm^−1^) (Javier‐Astete et al., 2021) at both growth stages further suggests that ROS‐dependent oxidative polymerization of lignin/phenolics occurred at both growth stages in AGS429 and UVE14 and led to cell wall re‐enforcement and stiffening. Similarly, cellulose accumulation occurred under DH stress in Arabidopsis (Leal et al., 2022). The substantial holocellulose decline at pod‐filling in UVE17 implies that even though this cultivar may possess sufficient lignin and phenolic content, cellulose/hemicellulose cross‐linking with lignin/phenol did not occur due to low pentose/hexose carbohydrates (1000 cm^−1^) and poor cellulose synthesis under DH stress, contributing to the cultivar's susceptibility to DH stress.

## 5 CONCLUSION

This study revealed the distinct DH stress tolerance mechanisms among edamame cultivars AGS429, UVE14, and UVE17. AGS429 responds to DH stress through reduced membrane damage and electrolyte leakage, helping to maintain cellular integrity. UVE14 predominantly relies on AsA for ROS scavenging, while UVE17 has weak capability for managing ROS, resulting in notable membrane damage (high MDA level) and electrolyte leakage. Interestingly, the reduction of ROS and membrane stability in AGS429 and UVE14 under DH stress was linked to increased cellulose and hemicellulose content. At the same time, the lack of ROS regulation in UVE17 led to a significantly reduced cellulose content at pod‐filling. Since CSC is in the membrane, membrane peroxidation could hinder cellulose synthesis in UVE17, reducing cellulose content. Overall, an effective antioxidative system in AGS429 and UVE14 under DH stress prevented oxidative damage, membrane peroxidation, and leakage, promoting cellulose accumulation and reinforcing cell wall integrity. Identifying the biochemical traits linked to DH stress tolerance and selecting resilient edamame cultivars (AGS429 and UVE14), are important for breeding DH stress‐tolerant varieties. This effort will support the farming industry in adapting to climate change. Future studies should examine how these biochemical responses affect edamame yield under DH stress to determine whether these traits indeed contribute to DH tolerance in UVE14 and AGS429.

## AUTHOR CONTRIBUTIONS

Jeremiah M. Hlahla performed experiments and prepared manuscript. Research design, Reactive oxygen species (ROS) and antioxidant systems research, funding acquisition, supervision, project administration, review and editing by Makoena J. Moloi. Guidance and supervision on cell wall biomass composition research by Mpho S. Mafa. Trial design and guidance on data analysis by Rouxlene van der Merwe.

## FUNDING INFORMATION

This research was funded by the National Research Foundation's (NRF) Thuthuka program (grant number TTK180502325292), NRF's African Coelecanth Ecosystem Programme (ACGR) partial cost scholarship (PCS, MND210514601451).

## Data Availability

All data is contained within the article, therefore data sharing to this article is not applicable.
